# Joint learning for improvement – interprofessional competence development within the framework of a co-operative project between the University of Applied Sciences for Health Professions Upper Austria and the Medical Faculty of Johannes Kepler University Linz

**DOI:** 10.3205/zma001539

**Published:** 2022-04-14

**Authors:** Christina Rinnhofer, Katharina Steininger-Kaar, Emil Igelsböck, Daniela Hochstöger, Sylvia Öhlinger

**Affiliations:** 1 University of Applied Sciences for Health Professions Upper Austria, Head of Competence Centre Learning and Interprofessionalism, Linz, Austria; 2 Johannes Kepler University Linz, Medical Faculty, Head of Center for Medical Education, Linz, Austria; 3 University of Applied Sciences for Health Professions Upper Austria, Head of Bachelor Programme Physiotherapy, Linz, Austria; 4 Johannes Kepler University Linz, Medical Faculty, Head of Curriculum Coordination, Center for Medical Education, Linz, Austria; 5 University of Applied Sciences for Health Professions Upper Austria, Head of University Development, Head of the Council, Linz, Austria

**Keywords:** interprofessionalism, project description, curriculum development, competency-based education

## Abstract

**Objectives:**

This project carried out in cooperation between the University of Applied Sciences for Health Professions Upper Austria (UASHPUA) and the Medical Faculty of Johannes Kepler University Linz (MFJKUL), describes the feasibility, i.e., the planning and implementation, and presents selected results of an inter-university lecture on interprofessional cooperation.

**Methodology:**

The lecture “Grundlagen zu interprofessioneller Zusammenarbeit im Gesundheitswesen (IPZ3I)”/“Introduction to interprofessional cooperation in health care (IPZ3I)” as well as an interprofessional job shadowing were designed. The pilot lecture started in the winter semester (WS) 2019/20. An evaluation of IPZ3I was undertaken by means of a questionnaire.

**Results:**

IPZ3I was held in the WS 2019/20 for 296 students from nine different health care professions and included a specialist lecture, the presentation of the professions, and interprofessional case processing. In the evaluation approx. 80% of the students described a better understanding of the interprofessional collaboration. More than 70% regard interprofessional courses in education as important or very important. The majority of respondents indicate that after completing the lecture they can make recommendations for action for interprofessional cooperation.

**Conclusions:**

The joint lecture IPZ3I will be maintained at both universities. The process of evaluation and adaptation of curricula at UASHPUA is currently underway. This includes, for instance, consultations with the curriculum officers at MFJKUL, and the exploration of further possibilities to identify and to implement joint interprofessional teaching aspects in the curricula. This shall be achieved by considering the existing resources, increasing student numbers at MFJKUL as of 2023, planned curricula revisions as well as using possible synergies aiming at an extension of the existing cooperation.

## Zusammenfassung

**Zielsetzung:**

Das vorliegende Projekt wird in Kooperation der Fachhochschule Gesundheitsberufe Oberösterreich (FH Gesundheitsberufe OÖ) und der Medizinischen Fakultät der Johannes Kepler Universität Linz (JKU) durchgeführt und beschreibt die Machbarkeit, d.h. die Planung und Umsetzung sowie stellt ausgewählte Ergebnisse einer hochschulübergreifenden, interprofessionellen Lehrveranstaltung dar.

**Methodik:**

Die Lehrveranstaltung LV „Grundlagen zu interprofessioneller Zusammenarbeit im Gesundheitswesen (IPZ3I)“ sowie ein interprofessionelles Shadowing wurden konzipiert. Start der Pilot-Lehrveranstaltung war im Wintersemester (WS) 2019/20. Eine Evaluierung von IPZ3I wurde mittels Fragebogen durchgeführt und ausgewertet.

**Ergebnisse:**

IPZ3I fand im WS 2019/20 für 296 Studierende aus 9 Gesundheitsberufen statt und inkludierte Fachvortrag, Präsentation der Professionen sowie interprofessionelle Fallbearbeitung. In der Evaluierung beschrieben ca. 80% der Studierenden ein besseres Verständnis für die interprofessionelle Zusammenarbeit. Über 70% sehen das interprofessionelle Angebot in der Ausbildung als wichtig oder sehr wichtig an. Der Großteil der Befragten gibt an, nach der LV Handlungsempfehlungen für interprofessionelle Zusammenarbeit geben können.

**Schlussfolgerung:**

Das Angebot der gemeinsamen Lehrveranstaltung IPZ3I wird an beiden Hochschulen beibehalten. Aktuell wird an der FH Gesundheitsberufe OÖ der Prozess der Evaluierung und Anpassung der Curricula eingeleitet. U. a. werden dazu Abstimmungen mit den Curriculumsverantwortlichen der Medizinischen Fakultät der JKU geführt und weitere Möglichkeiten zur Implementierung und Verortung gemeinsamer interprofessioneller Lehre in den Curricula geprüft. Dies erfolgt unter Berücksichtigung vorhandener Ressourcen, der erhöhten Studierendenzahlen an der Medizinischen Fakultät ab 2023, geplanten Curriculumsüberarbeitungen sowie Nutzung möglicher Synergien mit dem Fokus, die bestehende Kooperation auszubauen.

## 1. Introduction

Different initiatives cater for the health care professions’ demand for interprofessional cooperation at the working place through appropriate education and training. Thus, a contribution to a safe patient-centred and community-based health care system shall be achieved [1], [2], [3], [4]. A prerequisite for this is an agreement on education embracing values or attitudes in particular towards interprofessionalism as vital aspects of the teaching mission reflecting in the acquistion of knowledge and expertise [5], [6]. In this context, interprofessional education and cooperation are considered success factors for quality, safety and a positive outcome in health care [2], [7], [8].

The starting point for this is the successful development and sustainable implementation of interprofessional teaching in the education programmes of all the health care professions involved. This requires the creation of the appropriate structural framework of conditions and a long-term institutional approach to align the different education systems [3], [9]. It is crucial for universities to continuously improve their organisation and create suitable structures in order to integrate interprofessional teaching sustainably and mandatorily at their various training locations [10]. 

Interprofessional learning in education and training is promoted at UASHPUA and MFJKUL [11]. At UASHPUA 23 ECTS of the bachelor study programmes are credited to interprofessional and/or interdisciplinary learning in different settings. Founded in 2014, MFJKUL offers Bachelor and Master degree programmes in medical education. The curricula are built on problem- and case study-based approaches with high practical relevance. 

From the outset, both universities placed interprofessionalism in the focal point of their activities. It can be found in the competence areas of the educational regulations for Health Care and Nursing, medical laboratory and technical services (i.e., Biomedical Science and Radiological Technology), and Midwifery [https://www.ris.bka.gv.at/eli/bgbl/II/2008/200], [12], [13]. In addition, interprofessionalism and implications derived for education and training are reflected in the competence profiles of the health care professions which were developed based on the CanMed model by Frank & Danoff [14], for e.g. Physiotherapy and Occupational Therapy [15], [16]. § 1 in the competence profile of the bachelor curriculum for medicine at MFJKUL states: “Apart from expertise […] the skills to cooperate with […] other health care professionals […] will be developed.“ Thus, in the key course element “Cognitive skills and competences“ the following training objective is defined: “skill to critically assess medical data, to examine and to connect them with knowledge from other areas and to develop creative solutions“ [17].

The significance of interprofessionalism is also stressed in post-graduate, pioneering efforts regarding the development and further extension of primary care in Austria [18], and is referred to by the *National Catalogue of Learning Objectives in Medicine* (NKLM). NKLM focuses on the physician and describes competences which allow him or her “to work collaboratively, respectfully and effectively with many different scientific disciplines and other health care professions“ [http://www.nklm.de]. To ensure the acquisition of these competences, interprofessional education is an essential prerequisite for interprofessional cooperation and practice, and simultaneously, interprofessional teaching and learning form the basis for an effective, efficient, safe and sustainable health care provision [19], [20]. Thus, some education and training programmes in health care should be offered interprofessionally [21].

The aim of this project is to describe the feasibility, i.e., the planning, implementation and evaluation of an inter-university, interprofessional lecture, and the presentation of selected evaluation results. 

## 2. Methodology

Gradually developing interprofessional skills as part of the further development of the curricula of both universities is the basis and medium-term goal of the cooperation, which has been practiced since 2017 through regular exchange on development, planning and implementation.

In concrete terms, the cooperation started in 2018 with the planning of both the lecture “Grundlagen zu interprofessioneller Zusammenarbeit im Gesundheitswesen (IPZ3I – 0.5 ECTS)” and an interprofessional job shadowing at Kepler Universitätsklinikum, in which students could observe new health care work settings under the instruction of well-experienced course supervisors (SHA3U – 0.5 ECTS). IPZ3I was held as a day event. The dates for the job shadowing were individually appointed and implemented during the semester in accordance with the course supervisors. Both courses are mandatory for the students at MFJKUL. For the students at UASHPUA the study programmes in charge optionally accredit 0.5 ECTS, i.e., the heads of degree programmes accredit the course individually to appropriate lectures in the relevant curriculum. 

The planning team for the joint lecture which discussed and determined the scope, objectives and possibilities for implementation comprised members from both universities from various professions who teach about interprofessionalism as experts in their fields. Students were not involved in the planning of the pilot lecture due to time and administrative constraints. The results of the evaluation by the students and the recommendations from the teaching experts on interprofessionalism will feed into the further development of the lecture. To win the widest possible cooperation by the students, the lecture was designed to be student-centred, case-based, and competence-oriented, and incorporated teaching and learning formats delivering the learning approach of theory, exchange and observation [http://www.nklm.de]. All the study programmes involved were in their third semester to ensure a comparable educational background. The design was completed in spring 2019, and the pilot lecture started in the winter semester 2019/20.

The competence-building approach is oriented towards the six categories or rather objectives of the BC *(British Columbia) Competency Framework for Interprofessional Collaboration* [22]. This is achieved by embedding interprofessional communication and conflict-solving strategies in the assignments of the jointly offered lecture. The exploration of the subject “Functioning as a team”, the roles and responsibilities of the participating health care professions as well as the creation of opportunities for joint decision-making serve inter alia to reach for continuous improvement in quality. Its orientation towards recommended actions referring to aspects of interprofessional education (IPE) such as time, process, scheduling, and emotional control includes sufficient time for joint learning and reflection processes, as well as the possibility to repeatedly provide encounters, exchange and joint learning opportunities throughout the course of the training programme, and thus, inter alia, to reflect on opinions, values and attitudes regarding interprofessionalism. An ideal master plan for this includes various activities and learning experiences within the process of the trainings [21], [23].

For organisational matters the one-day lecture comprising 0.5 ECTS credits was offered to three groups of 100 students each in different locations at UASHPUA. A team of four lecturers from both universities was assigned to each of the groups. At first, the students worked on the significance and benefits of interprofessional cooperation. An initial 45-minute lecture in plenary with the focus on interprofessional education and practice provided a common knowledge base. Then the students, in groups of 10-15 each, learned on the basis of their previously prepared “show and tell sessions on professions” about different health care professions. Concrete assignments afforded the students the opportunity of applying occupation-specific skills in up to six different professions. Finally, this was followed by an exchange of interprofessional experiences by working on a case study in small groups of approx. 10 persons each to reflect on their own knowledge and competences, to network with other participants and to derive recommendations for action for interprofessional cooperation. The group work was chaired by students who were specifically trained for this purpose, and focused on cross-curricular learning objectives such as communication, team and conflict work. Each study programme designed a case study which was both discussed in the small specialist group and in the interprofessional group (for an overview of the course planning see attachment 1 ). 

An evaluation for the purpose of quality assurance and further development was carried out by a self-developed questionnaire survey. The survey was anonymous and conducted in writing by *LimeSurvey*. Following the lecture, all the students (N=296) enrolled in the WS 2019/20 were invited to assess the lecture after attendance (for an overview of the questionnaire see attachment 2 ). The analysis was performed using IBM SPSS Statistics 26. The majority of the questions had to be answered by ticking numbers from 1 (low) to 5 (high) based on the Likert Scale, including n/a answer choice. Group differences in terms of opinions as well as knowledge/skills regarding interprofessionalism and the various professions were determined by the Chi-square test. 

Students could make additional comments on the lecture in a free-text field. A first verification of the results was performed, an extensive qualitative assessment and a publication are planned at some other point. The evaluation results were presented to the lecturers within the scope of a focus group. In this context the lecturer responses were collected and recorded. The feedback was fed directly into the planning of the next academic year. For this purpose, a core group provided suggestions for lecture modification and further development and presented them to the entire group of lecturers. This process was followed by reflection, coordination and the determination of follow-up procedures. 

## 3. Results

IPZ3I lecture was offered in the WS 2019/20 to 296 third-semester students enrolled in Biomedical Science (N=20), Dietetics (N=18), Occupational Therapy (N=32), Healthcare and Nursing (N=86), Midwifery (N=22), Speech and Language Therapy (N=18), Medicine (N=66), Physiotherapy (N=23), and Radiological Technology (N=11) who at this stage of learning already demonstrate adequate expertise and communicative skills as prerequisites for case processing. This cohort comprised all the third-semester medical students and students from the respective bachelor programmes at UASHPUA from Linz. The lecture consisted of three components:

1. Knowledge acquisition on interprofessionalism,
2. learning intensively about other health care professions, including their professional profiles and work routines,
3. problem-based learning illustrated by practical casework.

The response rate for correctly completed questionnaires was 72% (213 responses). Overall the general evaluation results on organisational aspects will be dealt with separately and are not part of this project report. The results from the descriptive statistics on the items regarding interprofessionalism are illustrated in table 1 (Tab. 1).

The lecture was perceived very positively by the students. 77.25% (N=210) of the students stated that the seminar is relevant for their professional career and 79.15% (N=210) indicated that they gained a better understanding of interprofessional cooperation through the lecture. While the majority of the examinees, i.e., 86.26% (N=210), are able to make recommendations for action for interprofessional settings after attending the lecture, 70.79% (N=215) rate interprofessional training courses as important or very important. No significant connections between the opinions and knowledge/skills in relation to interprofessionalism and the relevant health care professions were established. Even the merging of the variable “study programme” with the categories diagnostic professions, medicine, midwifery, therapeutic professions and health care and nursing did not show significant differences. 

Annotations to the survey which were made by the students in free-text fields describe references to topics such as appreciation, essential prerequisite knowledge, the position of lectures on interprofessionalism in the curriculum or ideas to optimise administrative processes. It is planned to perform a structured analysis of the responses by following qualitative content analysis methods such as, e.g., the Mayring approach, and publish the results.

## 4. Discussion

As of the academic year 2019/20, IPZ3I has been the first inter-university interprofessional lecture for all third-semester students at MFJKUL and the students from eight bachelor programmes at UASHPUA. In this approach to interprofessional learning students are supported in developing their abilities to work independently while steering towards *21^st^* *Century Skills* [24]. In particular the course programme therefore includes interprofessional activities which are subject to mutual acquaintance and trust-building [25]. 

The lecture is mandatory at MFJKUL and is attended by all students (180 students at full capacity). The optional accreditation of the lecture by UASHPUA for health professions students shall be viewed critically in terms of its extra efforts in planning and implementation, albeit this course design allowed for a quick start of the interprofessional cooperation between the universities. In view of further curriculum developments at UASHPUA discussions are being held on arranging training courses to become part of the regular third-semester programme. However, restrictions on participation may occur in the future depending on the competence profiles of the various health care professions and the individual interests of the students. 

Evaluation instruments in use at the universities could not adequately capture the relevant aspects of interprofessional lectures. Thus, an individual questionnaire was developed to gain new impetus for the revision of the lecture. The current development of the lecture incorporates the students’ views on the basis of their evaluation. The potential for the improvement of interprofessional lectures deriving from the students’ involvement is illustrated by Schwarzbeck et al. [26]. The examination of the future use of relevant instruments such as, for instance, UWE-IP [27], the use of further mixed-method longitudinal studies as recommended by the Institute of Medicine [28] as well as the pre-post-evaluations for an effective further development is underway. The student evaluation results were presented to the lecturers in a focus group. With this in mind, the lecturer responses and perspectives were collected and included in the further development of the lecture [29]. In addition, the exchange, reflection and consultation with the group served to ensure the consistent quality of the course programme. The evaluation shall involve all the relevant stakeholders and be carried out at both qualitative and quantitative levels [30]. 

The results from test items on interprofessionalism used in the questionnaire relate to a positive student perception of the interprofessional education, emphasising in particular the benefits of learning about other health care professions. No significant connections between opinions and knowledge/skills in relation to interprofessionalism and the relevant health care professions were established. This corresponds to the results from other studies [31] which highlight that interprofessional learning promotes new aspects in education and training through direct contact and personal exchange with another health care profession, which otherwise could not have been better acquired [32]. Contrary to this, certain aspects were emphasised more strongly by medical students in interviews [33], and there are indications that they particularly benefit from content-related training in health care and nursing. Woermann et al. [34] showed that attitudes towards and the willingness to learn and work interprofessionally increase through knowledge about one’s own and other health care professions. 

The further development of interprofessional education is carried out inter alia through an orientation towards recommendations for action [23]. To ensure a gradual acquisition of interprofessional competences as required in the educational regulations, the continuous implementation of interprofessional cooperation in the study programmes through lectures and also across the curriculum is necessary. The lecture described above is a potential course for theory-, exchange- and action-based learning [23]. Moreover, it is an essential precondition for sustainable competence-building to continuously offer courses of various designs and structures throughout the study programme [23], and an appropriately adapted mixture of teaching and learning methods of different qualities is recommended [23].

The job shadowing is scientifically evaluated by an accompanying study. It is planned to disseminate the results in a publication. The universities continue to exchange information on a regular basis to explore further possibilities for cooperation such as, for instance, working on joint skills training or developing and implementing an interprofessional education module [3], [21].

## 5. Conclusions

Existing recommendations for action and best practice examples serve as a basis for the joint further development of interprofessional competences at the Medical Faculty of Johannes Kepler University Linz and at the University of Applied Sciences for Health Professions Upper Austria. In order to use the potential and synergy effects of already existing curricula, pilot projects were designed and implemented, and possible next steps have been considered for further curricula improvements.

The creation of an interprofessional lecture addressing a high number of participants and its implementation into the context of the different structural framework conditions of the two universities with different education systems are challenging and require a readiness at all institutional levels, in principle, as well as good consultation between the project team and the study programmes for its successful implementation. The presentation of professions, and the resulting casework performed by the students, are a good first approach. The examination and selection of relevant standardised evaluation methods are envisaged and a pre-post-design to assess the interprofessional competence-building is under development. 

The processes concerning the evaluation and adaptation of curricula at the University of Applied Sciences for Health Professions Upper Austria are currently underway. A standardisation of the structural embedding of interprofessional lectures in the curriculum of the bachelor programmes is being planned. For this, discussions and consultations with curriculum officers at the Medical Faculty of Johannes Kepler University Linz are being held and opportunities for the further identification and implementation of joint interprofessional teaching aspects in the curricula are being explored. This takes into account the existing resources, the increasing number of students at the Medical Faculty of Johannes Kepler University Linz as of 2023, the envisaged curriculum reviews and the use of synergies which aim at further intensifying the existing cooperation. 

## Data

Data for this article are available from the Dryad Digital Repository: [https://doi.org/10.5061/dryad.8sf7m0cn7], [35].

## Competing interests

The authors declare that they have no competing interests. 

## Supplementary Material

Lecture “Interprofessional cooperation in health care professions”

Questionnaire on the evaluation of IPZ3I

## Figures and Tables

**Table 1:**
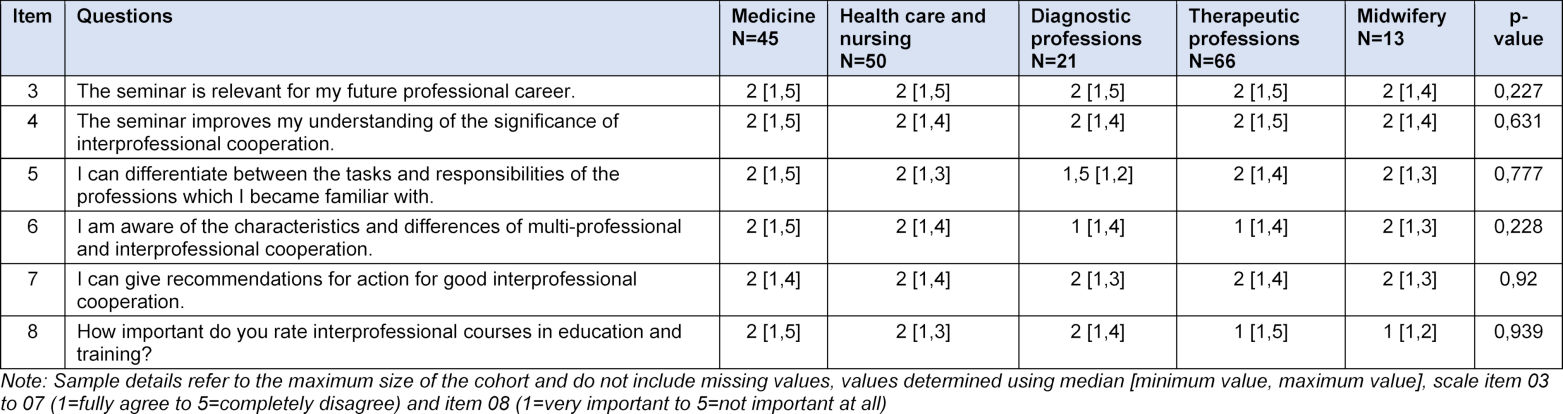
Descriptive statistics on items regarding interprofessionalism
