# Supplementary material for: Joint learning for improvement – interprofessional competence development within the framework of a co-operative project between the University of Applied Sciences for Health Professions Upper Austria and the Medical Faculty of Johannes Kepler University Linz
Source: GMS J Med Educ. 2022 Apr 14;39(2):Doc18. doi: 10.3205/zma001539 (PMC9174073; doi:10.3205/zma001539)
Supplement: Lecture “Interprofessional cooperation in health care professions” [file JME-39-18-s-001.pdf]

Attachment 1: Lecture “Interprofessional cooperation in health care professions”

Nr.	Topic	Time	Content	Learning objectives	Methods/Tasks/Arrangement/ Progress monitoring
1	Introduction	08:30-08:35	Outline of the lecture	Lectures, learning outcomes, organisational matters, framework of conditions, methodology	Lectures
2	Part 1:<br>Interprofessional cooperation in health care professions	08:35-09:20	What does interprofessional cooperation mean?	The students can define interprofessional cooperation.<br>The students have a common understanding/knowledge base of IPP/IPE.	Joint lecture (including physicians)
3	Part 2:<br>“Show and tell” sessions on professions/description of roles	09:20-11:30	Learning from and about each other (incl. 15-min. break)<br>• Which roles?<br>• Which responsibilities?<br>• Which tasks?<br>• Common interface in professional cooperation?<br>• Identify characteristic peculiarities<br>• Professional self-perception and professional perception by others	The students know other health care professions and can identify their responsibilities and core competences.<br>The students recognise similarities in and differences between health care professions.	“Show and tell” sessions on health care professions with application of practical examples prepared by students for students.<br>An even distribution of students in “show and tell” sessions (approx. 10 persons each).
4	Meet the team	11:30-11.55	Introduction to further steps<br>Meet the team members<br>Presentation of the case studies in the team and Q&A		Hosted by moderator
5	Part 3:<br>Case processing	11:55-12:25	Case analysis in the groups of health care professionals<br>Develop problem-objective-measures-plan (POM-plan)	The students work on the specific objectives for patient treatment according to their health care professions on the basis of a given case study.<br>The students derive measures for interprofessional work in their profession-specific groups.	Casework in small groups of students from the relevant study programme (approx. 2 to 4 persons each).
6	Part 3:<br>Case processing	12:25-13:00	Group presentations of POM plans from the health professions working groups<br>Adaptations to/corrections of the results by other health care professionals and lectures<br>→ outline joint objectives for patients	The students in an interprofessional team are able to explain and to represent the tasks/key activities and priorities of their own health care professions and others on the basis of a case study.<br>The students know professional interfaces.	Casework in the interprofessional groups (approx. 10 persons each) + host

Attachment 1 to Rinnhofer C, Steininger-Kaar K, Igelsböck E, Hochstöger D, Öhlinger S. *Joint learning for improvement – interprofessional competence development within the framework of a co-operative project between the University of Applied Sciences for Health Professions Upper Austria and the Medical Faculty of Johannes Kepler University Linz.* GMS J Med Educ. 2022;39(2):Doc18. DOI: 10.3205/zma001539

Nr.	Topic	Time	Content	Learning objectives	Methods/Tasks/Arrangement/ Progress monitoring
7	Part 3:<br>Case processing	14:00-14:50	Identify recommendations for action based on the case study on interprofessional cooperation in the relevant settings.	The students can develop and derive recommendations for action for high quality patient treatment through interprofessional cooperation on the basis of a case study.<br>The students are able to explain problem areas arising from the cooperation of different health care professions in patient treatment, they know the conditions for good interprofessional cooperation among the health care professions and can discuss them.	Identifying recommendations for action in interprofessional groups (approx. 10 persons each) + host<br>→ photo log on Moodle
8	Part 3:<br>Case processing	14:50-15:30	Preparation of walking tour<br>Reflection on the working process (incl. 15-min. break)	The students can reflect upon their own attitudes and their own resulting actions in interprofessional situations.<br>The students are aware of the conflict potential in interprofessional cooperation and can deal with it productively.	Reflection in small groups
9	Part 3:<br>Case processing	15:30-16:00	Walking tour:<br>• Presentation of settings<br>• Presentation of casework<br>• Presentation of the recommendations for action<br>• Presentation of the requests to other health care professionals regarding good cooperation	The students know examples of good practice and can derive concrete measures for their own health care professions.<br>The students learn about various interprofessional settings within the scope of the walking tour.	Presentation of results in the form of a walking tour<br>Photo-documentation of results<br>Casework in interprofessional teams<br>Moderation and poresentation
10	Conclusion	16:00-16:30	Wrap up and future prospects including evaluation		Summary of results by lecturers<br>LimeSurvey evaluation during lecture
