# Supplementary material for: Joint learning for improvement – interprofessional competence development within the framework of a co-operative project between the University of Applied Sciences for Health Professions Upper Austria and the Medical Faculty of Johannes Kepler University Linz
Source: GMS J Med Educ. 2022 Apr 14;39(2):Doc18. doi: 10.3205/zma001539 (PMC9174073; doi:10.3205/zma001539)
Supplement: Questionnaire on the evaluation of IPZ3I [file JME-39-18-s-002.pdf]

# Attachment 2: Questionnaire on the evaluation of IPZ3I

**Dear student! By answering the following questions, you are making an important contribution to the further development of**

## Interprofessionalism

| | I fully agree | I agree | neutral | I rather disagree | I do not agree at all | n/a |
|---|---|---|---|---|---|---|
| The seminar is relevant for my future professional career. | o | o | o | o | o | o |
| The seminar improves my understanding of the significance of interprofessional cooperation. | o | o | o | o | o | o |
| I can differentiate between the tasks and responsibilities of the professions I became familiar with. | o | o | o | o | o | o |
| I am aware of the characteristics and differences of multi-professional and interprofessional cooperation. | o | o | o | o | o | o |
| I can give recommendations for action for good interprofessional cooperation. | o | o | o | o | o | o |

## General questions on the lecture

| | I fully agree | I agree | I partly agree | I rather disagree | I do not agree at all | n/a |
|---|---|---|---|---|---|---|
| The spatial conditions (room size, acoustics, light, noise level, etc.) allow the participants to concentrate better on the course. | o | o | o | o | o | o |
| The number of participants was appropriate for the lecture. | o | o | o | o | o | o |
| The learning objectives were clearly outlined. | o | o | o | o | o | o |
| The content of the lecture corresponded with the learning objectives. | o | o | o | o | o | o |
| The organisation and the implementation of the lecture were good. | o | o | o | o | o | o |
| The delivery of the seminar was scheduled appropriately within the course of the study programme. | o | o | o | o | o | o |
| My prerequisite knowledge was sufficient to follow the contents of the lecture. | o | o | o | o | o | o |

How important do you rate interprofessional courses in education and training? — Not at all ——— Very important

I have enrolled for the following study programme: selection list

Have you already performed an internship within the course of your current study programme? — yes — no — If so, then go to question

Did you learn about interprofessional cooperation within your internship? — yes — no

Further comments:

**Thank you for participating in this evaluation!**

Attachment 2 to Rinnhofer C, Steininger-Kaar K, Igelsböck E, Hochstöger D, Öhlinger S. *Joint learning for improvement – interprofessional competence development within the framework of a co-operative project between the University of Applied Sciences for Health Professions Upper Austria and the Medical Faculty of Johannes Kepler University Linz.* GMS J Med Educ. 2022;39(2):Doc18. DOI: 10.3205/zma001539
